# Diagnostic value of upper airway morphological data based on CT volume scanning combined with clinical indexes in children with obstructive sleep apnea syndrome

**DOI:** 10.3389/fmed.2023.1150321

**Published:** 2023-03-17

**Authors:** Yanmin Shi, Meilan Gu, Xin Zhang, Mengmeng Wen, Runhua Li, Yifei Wang, Chen Li, Xianli Wang, Ruiyun Yang, Xinguang Xiao

**Affiliations:** Department of Radiology, Zhengzhou Central Hospital Affiliated to Zhengzhou University, Zhengzhou, China

**Keywords:** CT volume scan, upper airway morphology, children, OSA, diagnosis

## Abstract

**Background and purpose:**

Early diagnosis is important for treatment and prognosis of obstructive sleep apnea (OSA)in children. Polysomnography (PSG) is the gold standard for the diagnosis of OSA. However, due to various reasons, such as inconvenient implementation, less equipped in primary medical institutions, etc., it is less used in children, especially in young children. This study aims to establish a new diagnostic method with imaging data of upper airway and clinical signs and symptoms.

**Methods:**

In this retrospective study, clinical and imaging data were collected from children ≤10 years old who underwent nasopharynx CT scan(low-dose protocol)from February 2019 to June 2020,including 25 children with OSA and 105 non-OSA. The information of the upper airway (A-line; N-line; nasal gap; upper airway volume; upper and lower diameter, left and right diameter and cross-sectional area of the narrowest part of the upper airway) were measured in transaxial, coronal, and sagittal images. The diagnosis of OSA and adenoid size were given according to the guidelines and consensus of imaging experts. The information of clinical signs, symptoms, and others were obtained from medical records. According to the weight of each index on OSA, the indexes with statistical significance were screened out, then were scored and summed up. ROC analysis was performed with the sum as the test variable and OSA as the status variable to evaluate the diagnostic efficacy on OSA.

**Results:**

The AUC of the summed scores (ANMAH score) of upper airway morphology and clinical index for the diagnosis of OSA was 0.984 (95% CI 0.964–1.000). When sum = 7 was used as the threshold (participants with sum>7 were considered to have OSA), the Youden’s index reached its maximum at which point the sensitivity was 88.0%, the specificity was 98.1%, and the accuracy was 96.2%.

**Conclusion:**

The morphological data of the upper airway based on CT volume scan images combined with clinical indices have high diagnostic value for OSA in children; CT volume scanning plays a great guiding role in the selection of treatment scheme of OSA. It is a convenient, accurate and informative diagnostic method with a great help to improving prognosis.

**Highlights:**

– Early diagnosis of OSA in children is very important for the treatment.
– However, the traditional diagnostic gold-standard PSG is difficult to implement.
– This study aims to explore convenient and reliable diagnostic methods for children.
– A new diagnostic model was established combining CT with signs and symptoms.
– The diagnostic method in this study is highly effective, informative, and convenient.

## 1. Introduction

Obstructive sleep apnea (OSA) in children is a series of pathophysiological changes caused by frequent partial or complete upper airway obstruction during sleep that interferes with sleep architecture and ventilation in children (1–3). The prevalence of OSA in children is about 2%–4%, and it is on the rise (1). Its main clinical manifestations include sleep dyspnea/apnea habitual snoring, mouth breathing, sleep awakenings, daytime weakness and drowsiness. Habitual snoring is present in almost all children with OSA. Habitual snoring and sleep apnea are often the first symptoms, but are easily ignore (4). Without timely treatment, the long-term course of the disease can lead to a variety of complications such as developmental abnormalities, cognitive dysfunction, cardiovascular disorders, as well as mood disorders such as irritability, aggressive behavior, anxiety, and depression (5). Therefore, early identification, definite diagnosis and timely treatment are important to improve the prognosis of children with OSA.

At present, the clinical definite diagnosis of OSA in children depends on Polysomnography (PSG). The American Academy of Pediatrics (AAP) suggests that OSA should be screened in children as part of routine health maintenance. PSG should be performed for a definitive diagnosis in children with typical symptoms (e.g., snoring, sleep disturbance, or daytime hyperactivity) or risk factors (e.g., craniofacial, neurological, or genetic disorders) (6). However, it is difficult to implement PSG because of the long monitoring time, high cost, the need for the subject to sleep naturally throughout the night during the examination, and a well cooperation from parents and children. In addition, due to the lack of standard sleep monitoring conditions in some medical institutions, clinicians, especially pediatricians and ear, nose and throat (ENT) clinicians, diagnose the OSA mainly based on imaging examinations such as lateral cephalometric X ray films, CT scans of the head and neck, and clinical symptoms and signs of the child patients. Unfortunately, there is still a lack of satisfactory screening tools and objective scoring criteria in clinical practice yet (7). And consequently the accuracy of disease diagnosis much depends on the clinicians’ subjective cognition and experience of the disease. This paper aimed to explore a diagnostic method of combining upper airway morphological data with clinical symptoms and signs to predict OSA in children by analyzing the nasopharyngeal CT examination data and clinical data of children under 10 years of age with OSA.

### 1.1. Methods

**This is a retrospective study**. This study was approved by the Ethics Committee of Zhengzhou Central Hospital Affiliated to Zhengzhou University (approval number: 201978). The legal guardians of all subjects had singed the informed consents. All methods have been performed in accordance with the relevant guidelines and regulations.

### 1.2. Subjects

Clinical and imaging data were collected from children ≤10 years old who underwent nasopharynx CT scan at Zhengzhou Central Hospital Affiliated to Zhengzhou University from February 2019 to June 2020. The reasons for choosing the age range of the subjects are: adenoids develop rapidly after birth, reach their maximum size in early childhood, begin to regress around 8–10 years of age (8). In addition, OSA has a peak incidence around 2–8 years of age (9). Exclusion criteria: (i) nasopharyngeal cavity bleeding; (ii) immunodeficiency; (iii) previous history of turbinatectomy or adenoidectomy; (iv) nasopharyngeal or cervical masses or occupying lesions involving the upper airway; (v) skull base fracture or craniofacial malformation; (vi) chondrodysplasia, hypothyroidism and acromegaly; (vii) various central nervous system diseases.

## 2. Materials and methods

### 2.1. Imaging indexes and detecting methods

**Figure 1:**
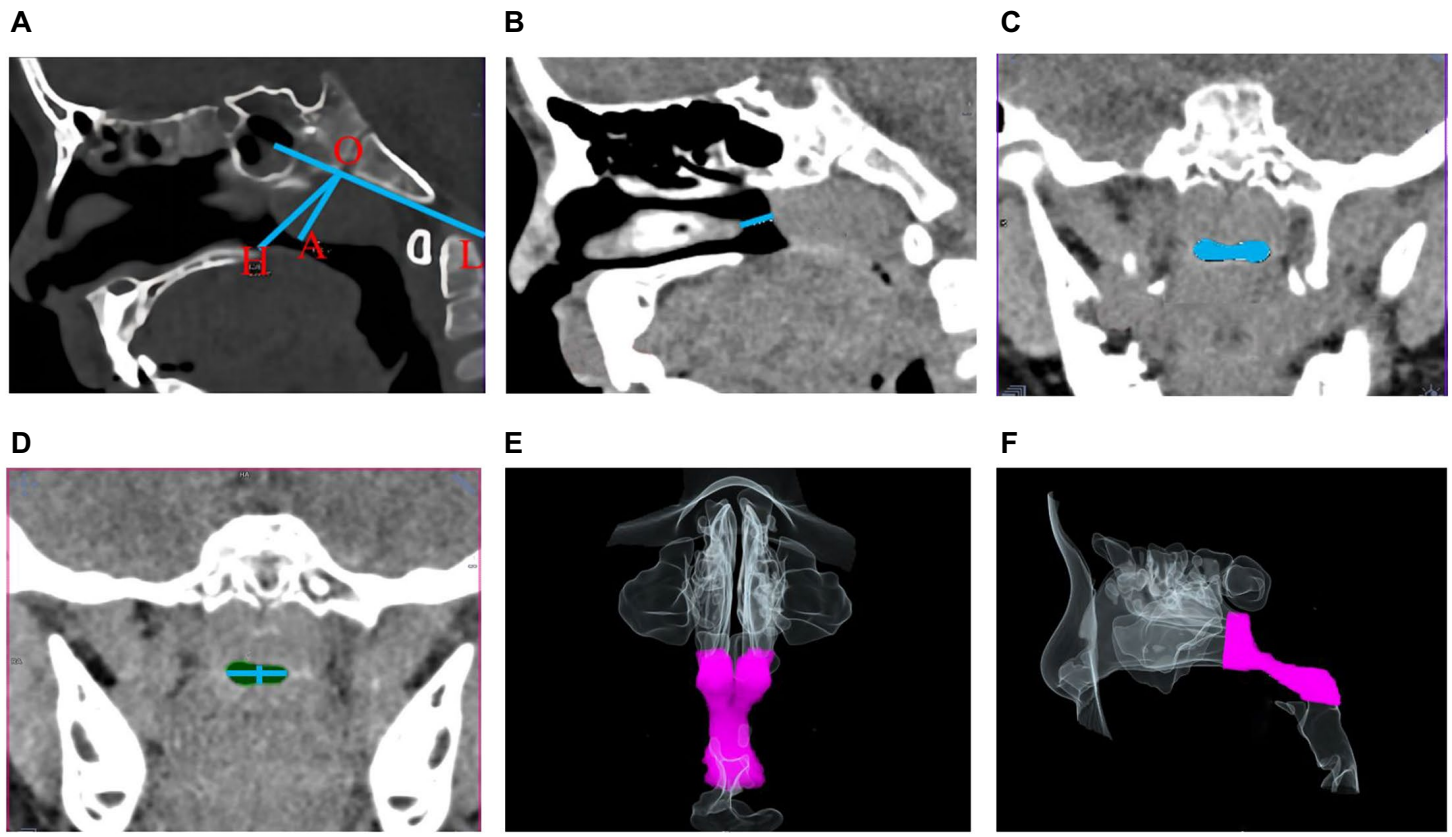
Schematic diagram of morphological indicators of the upper airway. **(A)** Nasopharyngeal cavity width (OH) and adenoids thickness (OA); **(B)** Right nasal gap size; **(C,D)** Minimum cross-sectional area of the upper airway in coronal position and its upper-lower, right–left diameters; **(E,F)** Coronal (E) and sagittal (F) upper airway volume model VR plots.

The measurement indexes and the corresponding methods are shown in Table 1; Figure 1.

**Table 1:** Measurement of morphological data of the upper airway based on CT volume scan images.

Measurement image source	Measurement indicators	Specific observations	Measurement methods	Reference illustrations
Median sagittal position: positive mid-sagittal images were obtained by positioning the upper edge of the soft and hard palate in the transverse axis position	N line	Nasopharyngeal cavity width	Distance from the lower border of the pterygo-occipital cartilage junction (point O) to the upper border of the soft and hard palate junction (point H) in line	Figure 1A
	A line	Adenoid thickness	Make an extension line (L) along the inferior border of the occipital bone and a vertical line of the extension line through the most convex point of the adenoids	Figure 1A
	Nasal gap	Mean of left and right posterior nostril gap sizes	Parallel inferior turbinate sagittal images measuring the distance from the posterior margin of the left and right inferior turbinates to the anterior margin of the adenoids	Figure 1B
Coronal images: sagittal image reconstruction of the coronal image *via* the most convex point of the adenoids, perpendicular to the long axis of the airway	Upper airway area	Minimum cross-sectional area of upper airway	Coronal image reconstruction *via* the most convex point of the adenoids, perpendicular to the long axis of the airway	Figure 1C
	Upper and lower airway diameters and right and left diameters	Upper airway minimum cross-sectional dimensions upper-lower diameter and left -right diameter	Coronal images were reconstructed *via* the most convex point of the adenoids, perpendicular to the long axis of the airway, and the size of the upper- lower and right–left trajectories of the airway at this level	Figure 1D
Original axial images: parallel inferior turbinate level	Upper airway volume	VR images and volume size of the nasopharyngeal cavity in the upper airway	The posterior border of the inferior turbinate was used as the anterior border, the parietal wall of the nasopharynx as the posterior superior border, and the level of the soft palate at the level of the uvula not connected to the oropharynx as the inferior border, and the area of interest was outlined along the edge of the airway layer by layer, then the volume was calculated by the post-processing software of the CT device workstation.	Figures 1E,F

**Figure 2:**
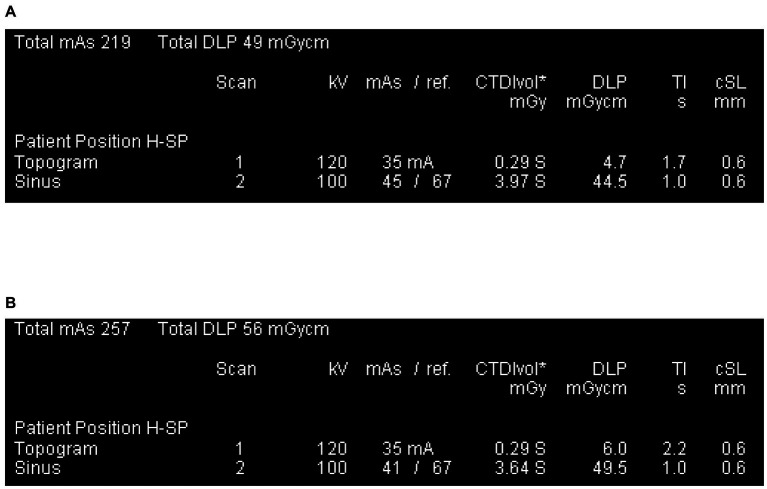
Patient protocol. **(A)** The smallest DLP across all study subjects, was obtained from a 1-year-old child. **(B)** The largest DLP of all study subjects, was obtained from a 10-year-old child.

All the imaging data were obtained from high-resolution CT volume scan images with a Siemens Somatom definition flash CT scanner, installed in June 2018.The detector combine with Siemens’ proprietary Ultra Fast Ceramics scintillator the SOMATOM Definition Flash acquires 2 × 128 slices per rotation at outstanding dose efficiency. Except for children under 3 years of age who breathe naturally, all the children were given a CT scan of nasopharynx when deep inhaling and breathy holding (the volume of nasopharynx is the largest at this time) in static and supine position. During scanning, all children were in supine position, with the audio-orbital line perpendicular to the examination bed surface, the midsagittal position of the head and body coincident with the table top. All children were fixed with head rest to avoid artifacts caused by head movement Cases with image artifacts such as swallowing and respiratory movement were excluded. The scanned part was the nasopharynx, from the upper limit of the nasopharyngeal apex to the lower limit of the epiglottis. All children were examined by low-dose scanning protocol: tube voltage 100KV, Care Dose 4D automatic tube current technology and iterative reconstruction algorithm, scanning layer thickness 1.0 mm, layer spacing 0.6 mm, scan length 11.2–13.6 cm, and scanning time 3-4S. The min-max dose length product(DLP)is 49-56 mGy.cm (see Figure 2 patient protocol). Each indicator was measured twice and averaged by two radiologists double-blindly. Paired *t*-test was used to test the consistency between two experts, showing good agreement (*t* = −1.716 ~ 1.916, *p* > 0.05). The mean of data measured by the two experts was used as the final value of the indicator.

Referring to the method in previous research (10), the original images were reconstructed into coronal and sagittal images by multi-planar reformation (MPR). Imaging of the upper airway was measured in the original transaxial and reconstructed coronal and sagittal images. VR images of the upper airway were reconstructed based on the original axial images by volume rendering (VR).

A/N represented the adenoid size. According to A/N, participants were classified into mild adenoid hypertrophy group(A/N ≥ 0.6) and severe adenoid hypertrophy group (A/N ≥ 0.7) (11).

### 2.2. Clinical indexes and evaluation methods

Collect clinical information of children, including whether it exists habitual snoring, mouth breathing, adenoidal facies, chronic sinusitis and secretory otitis media.

Chronic sinusitis, secretory otitis media and adenoid face were diagnosed according to the guidelines and consensus of imaging diagnostics experts (12–16).The information of clinical signs, symptoms and others were obtained from the medical records. Habitual snoring was categorized into <3 months and ≥ 3 months according to its duration. OSA was diagnosed by two ENT clinicians according to the diagnostic criteria and opinions of OSA published by the AAP and the American Academy of Sleep Medicine (AASM) (4, 17). Subjects were grouped into the OSA or non-OSA based on the diagnosis. When the two independent diagnoses of chronic sinusitis or secretory otitis media or adenoid face or OSA in the same patient were inconsistent, the two clinicians discussed and made a final diagnosis.

## 3. Sample size

Based on the diagnostic model, the sample size was calculated by the formula (1), where *α* = 0.05, allowable error *δ* = 0.15, and *p* is the sensitivity or specificity of the method to be tested. Previous findings (18) showed that the sensitivity and specificity of diagnosis on OSA with combined adenoid and upper airway morphology were 90.0% and 79.8%, respectively. The minimum sample size of the OSA group was 16, and that of the non- OSA group was 28. A margin of 20% was used for the sample size to account for any invalid samples. Therefore, the minimum sample size was 20 for the OSA group and 34 for the non-OSA group.


(1) $$n={\left(\frac{z_{\alpha }}{\delta }\right)}^{2}\left(1-p\right)p$$

### 3.1. Statistical analysis

Statistical analyses were performed with IBM SPSS Statistics 23.0 software. Single-factor unconditional logistic regression was used to screen the risk factors for OSA. Statistically significant variables were assigned values. For categorical data, each category was assigned a value based on the magnitude of the regression coefficient (0–5 points). For quantitative variables, logistic regression analysis was performed with the presence or absence of OSA as the dependent variable, and diagnostic probabilities were calculated, and quantitative variables were assigned a value (0–5 points) after transformed into categorical variables based on the diagnostic probability values. All indicators were scored and summed up. Receiver Operating Characteristic (ROC) analysis was performed with sum as the test variable and OSA as the state variable, and the optimal cut-off value of the sum was determined based on the Youden’s index. And the diagnostic efficacy was assessed in terms of area under the curve (AUC), sensitivity, specificity, and accuracy. All the above statistical tests were two-sided with test level *α* = 0.05.

## 4. Results

### 4.1. General characteristics of subjects

Based on the inclusion criteria, a total of 140 subjects imaging and case information were collected, out of which 2 and 8 subjects were excluded due to adenoidectomy and nasopharyngeal hemorrhage respectively, resulting in the inclusion of 130 subjects. There were 50 males and 80 females, aged 1–10 years with a mean age of 5.01 ± 2.19 years. There were 92 (70.8%) and 19 (14.5%) patients with chronic sinusitis and secretory otitis media, respectively. There were 25 patients in the OSA group with a proportion of 19.2%, including 17 males and 8 females with a mean age of 5.56 ± 2.20 years. There were 105 in the non-OSA group, 63 and 42 males and females, respectively, with a mean age of 4.88 ± 2.17 years. There was no statistical difference in age and gender distribution between the OSA and non-OSA groups (age: *t* = 1.410, *p* = 0.162; gender: *χ*^2^ = 0.546, *p* = 0.461).

### 4.2. Univariate logistic regression analysis of the relationship between clinical information and OSA

The results of logistic regression analysis with the presence or absence of OSA as the dependent variable are shown in Table 2. Five statistically significant variables were found: A-line, nasal gap, mouth breathing, adenoidal face and habitual snoring. The results showed that the risk of OSA increased 1.047-fold (95% CI 1.509–2.776) for each 1-mm increase in the A-line (adenoid thickness) and decreased 39.4% for each 1-mm increase in the nasal gap (OR = 0.606, 95% CI 0.475–0.774). The risk of OSA in mouth breathers was 7.778 times greater than in those without mouth breathing (OR = 7.778, 95% CI 2.223–27.217). The risk of OSA was 25.000 times higher in those with adenoidal facies than in those without this sign (OR = 25.000, 95% CI 3.664–170.586). The risk of OSA was 50.500 and 168.333 times higher in those who snored for <3 months and ≥ 3 months, respectively, than in those who did not snore (*p* < 0.05).

**Table 2:** Univariate logistic regression analysis of the relationship between clinical information and OSA (*n* = 130).

variable	*β*	S.E.	Wald	OR	95% CI	*p*
Age	0.144	0.103	1.957	1.154	0.944–1.412	0.162
Upper airway volume	0.000	0.000	0.353	1.000	1.000–1.000	0.553
Upper airway area	−0.005	0.004	1.948	0.995	0.987–1.002	0.163
A line	0.716	0.155	21.242	2.047	1.509–2.776	<0.001
N line	0.044	0.098	0.207	1.045	0.863–1.266	0.649
Nasal gap	−0.500	0.125	16.128	0.606	0.475–0.774	<0.001
Male	0.349	0.473	0.543	1.417	0.561–3.578	0.461
Mouth breathing	2.051	0.639	10.303	7.778	2.223–27.217	0.001
Chronic sinusitis	0.074	0.494	0.023	1.077	0.409–2.837	0.880
Secretory otitis media	0.276	0.673	0.169	0.759	0.203–2.835	0.681
Adenoidal facies	22.721	28420.722	0.000	7.37*10^9^	--	0.999
Habitual snoring			37.887			<0.001
<3 months	3.922	0.788	24.769	50.500	10.777–236.633	<0.001
≥3 months	5.126	1.130	20.583	168.333	18.384–1541.355	<0.001

95% confidence interval for OR not calculated because the standard error is too large.

### 4.3. Assignment for variables

Based on the results of the univariate analysis, five statistically significant variables were included and assigned.

#### 4.3.1. Assignment for categorical variables

Values of categorical variables were assigned according to the magnitude of the regression coefficient. The reference group was assigned a score of “0”(all the regression coefficients needed to be greater than 0; if the regression coefficient was less than 0, the direction of the variable assignment was reversed for dummy variables). If there was no statistical difference between the category and the reference group, “0”was assigned. The other categories were rounded to the closest value according to the regression coefficient value as a score: “close 1” was assigned “1”; “close 2”was assigned “2,” and so on; if *β* ≥ 5, “5”was assigned. For example, those who sleep with mouth breathing were assigned “2,” and those without mouth breathing were assigned “0.” Although the logistic regression showed that the *OR* value for adenoidal facies contained 1, but considering that there were only 2 cases of adenoid facies in this study, which may result in a false negative error; and previous studies (16, 19, 20) have reported a significant association between adenoid facies and OSA, “5” was assigned to those with adenoid facies in the later analysis, otherwise “0” was assigned.

#### 4.3.2. Assignment for quantitative variables

Logistic regression analysis was performed and diagnostic probabilities were calculated with the presence or absence of OSA as the dependent variable. Decile splits were performed according to diagnostic probabilities, with probabilities 0.1, 0.2,0.3, 0.4,0.5 as splits.There were 6 groups with probability ranges of (0–0.1), [0.1–0.2], [0.2–0.3], [0.3–0.4), [0.4–0.5), and ≥ 0.5. The original quantitative variables were then grouped according to probabilities against the original quantitative variables, rounding up the original values to the closest and regrouping. The logistic regression was performed again with the new categorized ordinal variable as dummy variable. And each category were assigned according to the regression coefficients in the same way as for the categorical variables. The assignment of statistically significant variables in the univariate analysis is shown in Table 3.

**Table 3:** Assignments for clinical information.

variable	Original values	*n*	Assignments
A line(mm)	<12	62	0
	[12, 13.5)	28	1
	[13.5, 15)	14	2
	≥15	26	5
Nasal gap(mm)	≤2.5	8	3
	(2.5, 5.5]	42	2
	(5.5, 8.5]	54	1
	>8.5	26	0
Mouth breathing	No	118	0
	Yes	12	2
Adenoidal facies	No	128	0
	Yes	2	5
Habitual snoring	No	107	0
	< 3 months	12	4
	≥ 3 months	11	5

#### 4.3.3. Diagnostic effectiveness of sum of 5 variables on OSA

**Figure 3:**
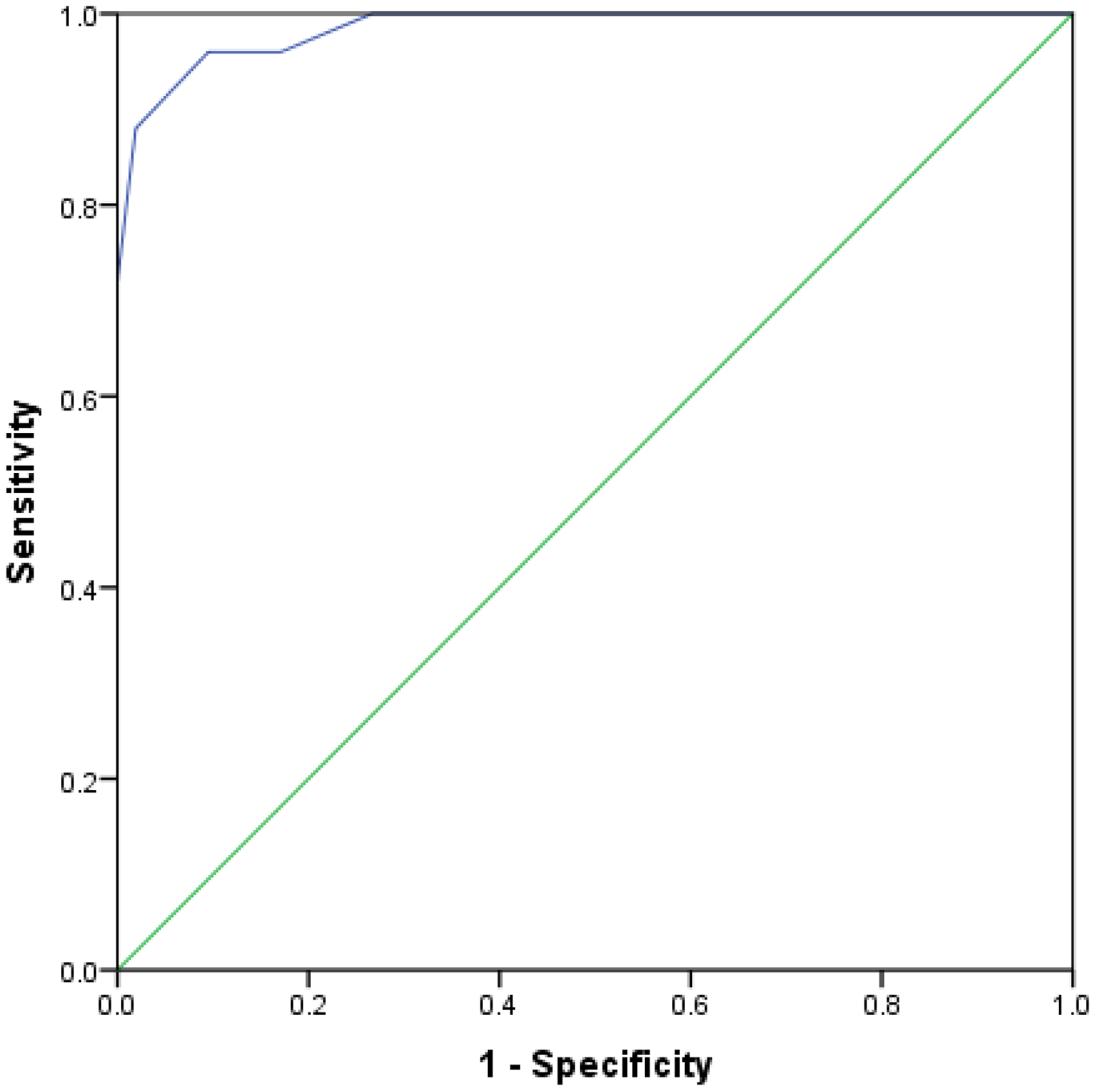
ROC curve for the diagnosis of sum on OSA.

The scores for the five variables of A-line, nasal gap, mouth breathing, adenoidal facies and habitual snoring were summed up. Here, we simply call it “ANMAH score.” ROC analysis was performed and the curves were plotted with sum as the test variable and OSA as the status variable (Figure 3) to test the diagnostic efficacy of sum on OSA. The results showed that the AUC was 0.984 (95% CI 0.964–1.000). The diagnostic efficacy of the model at different cut-off points is shown in Table 4. When cut-off points is 7 (patient with ‘sum >7’ could be considered to have OSA), the Youden’s index reached the maximum, the sensitivity was 88.0%, the specificity was 98.1%, and the accuracy was 96.2%.

**Table 4:** The diagnostic efficacy of the model at different cut-off points.

Cut-off point	Sensitivity	Specificity	Youden’s index	Accuracy
>4	100.0%	73.3%	0.733	78.5%
>5	96.0%	81.9%	0.779	84.6%
>6	96.0%	89.5%	0.855	90.8%
>7	88.0%	98.1%	0.861	96.2%
>8	76.0%	99.0%	0.750	94.6%
>9	68.0%	100.0%	0.680	93.8%

## 5. Discussion

Early diagnosis is a prerequisite for reducing the complications and improving the prognosis of OSA. Though PSG is recognized as the gold standard for the diagnosis of OSA (7, 9, 21), for various reasons, it is difficult to implement in children, especially preschool-aged patients. Exploring a less costly, convenient and highly accurate method for the diagnosis of OSA in children that can replace PSG has been the goal of researchers in recent years. In this study, the method of combining CT volume scan data (adenoid thickness and posterior nasal gap size), with clinical indexes(mouth breathing, sleep habitual snoring symptoms and adenoid facial signs) was found to have high sensitivity, specificity, and accuracy (the sensitivity the specificity and the accuracy was 88.0%, 98.1%, and 96.2%, respectively**)** for the diagnosis of OSA, which could be a convenient and effective way to diagnose OSA in children. Moreover, CT volumetric scan can not only determine the site and degree of upper airway obstruction, but also evaluate the complications such as chronic sinusitis and secretory otitis media comprehensively, which can provide more information for the choice of OSA treatment plan.

**Other studies about the diagnosis of OSA in children and their defects**. At present, some other diagnostic models of OSA in children have limitations. Jing Zhang et al. (22) collected clinical data information of 136 habitual snoring children(1–12 years)and establish a diagnosis model of OSA with pediatric sleep questionnaire, minimum nocturnal transcutaneous oxygen saturation of pulse (SpO2) monitoring,age and neck circumference. Its sensitivity was 80.9% and specificity was 76.2%. However, the large age span of subjects in the study may affect the authenticity of the results, furthermore nocturnal SpO2 monitoring still needs to be performed all night, which is difficult to implement. Lai CC et al. (7) collected data of BMI Z-score, tonsil volume, updated Friedman tongue position, snoring visual analog scale, nasal obstruction and mouth breathing in children, and established a diagnostic model by calculating and analyzing apnea-hypopnea index (AHI). The sensitivity of its AHI ≥ 5 and AHI ≥ 10 for diagnosing OSA was 75.6% and 84.6%, while the specificity was 61.7% and 56.5%, respectively. The unsatisfactory diagnostic efficacy of model in this study may be related to the adenoid hypertrophy, an important factor to trigger OSA in children, was not included.

**Therefore, in an excellent diagnostic model pathophysiological mechanism should be considered**. Computerized tomography is often used to evaluate the upper airway in the research about pathophysiology of OSA. Several studies have explored their diagnostic potential (23). Van Holsbeke et al. (24) found that functional 3D imaging parameters from computed tomography (CT) scans of upper airway better predicted obstructive sleep apnea (OSA) severity than standard clinical markers in children. Some subjects in the study had a history of upper respiratory surgery, and whether this factor would affect the accuracy of the study results is unknown. Alsufyani et al. (25) tested the feasibility in using cone beam CT (CBCT) to analyze the nasal and pharyngeal airway space post-surgery with meaningful methods of analyses, and correlating imaging findings with clinical outcomes in children with SDB(sleep disordered breathing) symptoms and maxillary-mandibular disproportion. They found that using point-based analyses, new imaging airway measures including constriction and patency better explained changes in clinical symptoms compared to conventional measures. However, patients need to have a higher degree of cooperation in the process of CT examination in this study. Our study is different from theirs because we mainly focus on the convenient and reliable diagnosis of OSA in children. And our research has been able to meet the requirements of diagnosis.

It is just based on the comprehensive consideration of pathophysiology to select CT volume scan data of upper airway morphology as the model indexes in this study. Adenoid hypertrophy is a major causative factor for OSA in children (6, 9, 21, 23, 26, 27). It is reported that structural abnormality of the upper airway caused by adenoid hypertrophy is the major factor in the pathogenesis of OSA in children. Resection of hypertrophic adenoids can rapidly relieve upper airway obstruction, which remains the first-line treatment of adenoid hypertrophy-induced OSA (1, 4, 9, 26, 27). CT volume scan can rapidly obtain images of the nasopharynx of children, clearly display the morphological characteristics of the upper airway. It can not only provide detailed anatomical data of the axial, sagittal and coronal surfaces of the upper airway, but also determine whether pathogenic factors causing OSA were existed such as adenoid hypertrophy, pharyngeal space-occupying lesion, etc.

**Although the symptoms and signs of OSA are important, they must be combined with other objective criteria to diagnose OSA accurately**. Several studies (9, 21, 27) pointed out that the symptoms and signs of the disease should be paid much attention to because they are important basis for the initial diagnosis of OSA in children. This study also demonstrated that symptoms such as sleep with mouth breathing, habitual snoring and adenoid facies increased the risk of OSA exponentially. However, SLAATS et al. reported (23) that symptoms/signs alone or being combined were not satisfactory in diagnosing OSA and predicting therapeutic effect. The subjective assessment of adenoid size does not correlate significantly with OSA severity, while the objective measurement of adenoid volume can well determine the severity of OSA. A combination of clinical features with other diagnostic tools was recommended. The univariate analysis in this study revealed that adenoid thickness was positively associated with OSA, whereas nasal gap size was negatively associated with it, which is consistent with previous findings that OSA is almost always associated with adenoid hypertrophy without severe malformations or complications (23, 26). In this study, both the upper airway morphological data and clinical indicators were included. Based on CT volumetric scan image data, this study quantifies the morphology and size of upper airway structures combining with clinical symptoms and signs, which can scientifically and accurately evaluate the role of different indicators on the diagnosis of OSA in children.

**The CT scanning technology is advanced and the radiation used in this study is at a safe dose**. In recent years, the application of ultra-high-speed CT, advances in examination techniques and optimization of protocols have made it possible to obtain clear, large amounts of image data at lower radiation doses (28, 29). The min-max dose length product(DLP) on all subjects is 49-56 mGy.cm, below the internationally recommended reference levels for children aged 1,5,10 (50, 60, 100 mGy.cm) (30). CT volume scan is a fast, convenient and noninvasive examination that can be performed both during wakefulness and sleep and be available in most medical institutions.

**One of the advantages of this study Is the high diagnostic efficiency**. In this study, rich morphological data of the upper airway and clinical symptoms and signs were included. Finally, five important indicators were screened out by univariate analysis to establish a diagnostic model (ANMAH score), in which adenoid thickness (A-line), nasal gap, mouth breathing, adenoid facial appearance and habitual snoring were scored and summed up. When ‘sum>7’, the accuracy rate for OSA reached 96.2%. This method has high sensitivity and specificity for diagnosing OSA in children and is superior to previous studies in diagnostic efficacy (7, 22). In addition, the diagnosis by the ANMAH score is easy for clinicians to operate, which can exclude the interference of subjective factors and improve the stability of diagnosis greatly.

**The second advantage of this study is that CT scan can also provide much important information and diagnose complications, which is conducive to the choice of treatment plan**. Although adenoidectomy remains one of the primary treatments for OSA in children with adenoid hypertrophy, there is no definitive conclusion on whether adenoidectomy will affect children growth and development. Adenoids are immune tissue. There is still disagreement on whether adenoidectomy should be performed and the extent of resection (31). The 2012 edition of the AAP guidelines (4) noted that adenoidectomy was used as the primary treatment method in moderate and severe OSA children with adenoid hypertrophy and no surgical contraindication. However, endoscopic or imaging evaluation of the upper airway conditions (including the nose, nasopharynx, oropharynx, larynx, and throat) is required. Adenoidectomy is also written into the guidelines as an important treatment in several countries for the management of child secretory otitis media, especially in children with nocturnal habitual snoring and nasopharyngeal infections (32). Gulottal et al. (3) suggested that recurrent otitis media and adenoid hypertrophy should also be taken into account as an important influencing factor in the diagnosis and treatment of OSA disease. Therefore, individualized and precise diagnosis is a prerequisite for treatment (6, 26). Before making an appropriate treatment plan, doctors need to accurately master the patient’s disease-related information. For example, whether the upper respiratory tract is unobstructed, whether there is nasopharyngeal mass, whether there is chronic sinusitis and otitis media, etc. All this information can be obtained by volumetric CT scanning. In this study, 92 cases (70.8%) of chronic sinusitis and 19(14.6%) cases of secretory otitis media were detected from 130 subjects. This provides an important reference for the formulation of personalized treatment plan.

However, this study has some weakness. First, obesity is one of risk factors for OSA in children. OSA has a high prevalence in obese children. Some studies suggested that obesity and mild adenoid hyperplasia can cause another type of OSA (1, 33). Obesity was not included in this study as an indicator, so its role in OSA could not be distinguished. More attention on obesity needs to be paid in future studies. In addition, the nasopharynx CT of some children under the age of three is scanned when natural breathing, so the upper airway volume may be slightly smaller than that when deep inhaling and breathy holding, which may result in some bias. Finally, the sample in this study was derived from a single center and has not been confirmed in other countries or regions, therefore future verifying in multicenter studies with large sample is needed.

In conclusion, morphological data of the upper airway based on CT volume scan combined with clinical indicators are of high value in the diagnosis of OSA in children. It is simple, reliable, informative and easy to implement in most medical institutions. This method is expected to be of high benefit in the diagnosis and treatment of OSA in children.

## Data availability statement

The raw data supporting the conclusions of this article will be made available by the authors upon request. Requests to access these datasets should be directed to XX, xiaoxinguang126@126.com.

## Ethics statement

The studies involving human participants were reviewed and approved by This study was approved by the Ethics Committee of Zhengzhou Central Hospital Affiliated to Zhengzhou University (approval number: 201978). Written informed consent to participate in this study was provided by the participants’ legal guardian/next of kin.

## Author contributions

YS performed data collection, analysis and explanation, reviewed the literature, and wrote most of the manuscript. MG and XZ participated the suggestion of the concept, design of experimental suggestions of the research procedures. MW participated in the revision of the article. YW and CL made much contributions to data collection and analysis. RL, XW, and RY participated in the research procedures and made some data analysis and explanation. XX participated in the study conception, design, and reviewed the final manuscript. XX was responsible for the content of the manuscript and the integrity of the data analysis. All authors contributed to the article and approved the submitted version.

## Funding

This work was supported by Science and Technology Research Project of Henan Provincial Department of Science and Technology, Item No.: 212102310707.

## Conflict of interest

The authors declare that the research was conducted in the absence of any commercial or financial relationships that could be construed as a potential conflict of interest.

## Publisher’s note

All claims expressed in this article are solely those of the authors and do not necessarily represent those of their affiliated organizations, or those of the publisher, the editors and the reviewers. Any product that may be evaluated in this article, or claim that may be made by its manufacturer, is not guaranteed or endorsed by the publisher.

## Abbreviations

OSA: obstructive sleep apnea
PSG: Polysomnography
AAP: American Academy of Pediatrics
ENT: Ears, nose, and throat
DLP: Dose length product
MPR: Multi-planar reformation
VR: Volume rendering
AASM: American Academy of Sleep Medicine
ROC: Receiver Operating Characteristic
AUC: Area under the curve
SpO2: Oxygen saturation of pulse
AHI: Apnea-hypopnea index
